# Depression-like state behavioural outputs may confer beneficial outcomes in risky environments

**DOI:** 10.1038/s41598-019-40390-3

**Published:** 2019-03-07

**Authors:** Marco A. Vindas, Siri H. Helland-Riise, Göran E. Nilsson, Øyvind Øverli

**Affiliations:** 1 Department of Food Safety and Infection Biology, Faculty of Veterinary Medicine and Biosciences, Norwegian University of Life Sciences, Oslo, Norway; 2 Department of Biosciences, University of Oslo, Oslo, Norway

## Abstract

Recent theories in evolutionary medicine have suggested that behavioural outputs associated with depression-like states (DLS) could be an adaptation to unpredictable and precarious situations. In animal models, DLS are often linked to diverse and unpredictable stressors or adverse experiences. Theoretically, there are a range of potential fitness benefits associated with behavioural inhibition (typical to DLS), as opposed to more active/aggressive responses to adverse or uncontrollable events. This stance of evolutionary medicine has to our knowledge not been tested empirically. Here we address a possible key benefit of behavioural inhibition in a comparative model for social stress (territorial rainbow trout). By treating fish with the fast-acting antidepressant ketamine, we reversed the behavioural inhibition (*i*.*e*. stimulated an increase in activity level) in subordinate fish. During confrontation with a previously unfamiliar larger, aggressive and dominant individual, this increase in activity led to higher amounts of received aggression compared to sham-treated subordinates. This suggests that the behavioural inhibition characterizing animal models of DLS is indeed an effective coping strategy that reduces the risk of injuries in vulnerable social situations.

## Introduction

Depression is a major mood disorder often linked to diverse and unpredictable stressors and/or adverse experiences. Evolutionary-based medicine is an emerging field in which evolutionary theory is used to elucidate both ultimate and proximate explanations for diseases. However, from a standpoint of evolutionary and comparative biology, depression is a paradox. That is, depression is often associated with great disadvantages for survival and reproduction^1–3^, while also being highly heritable^4^ and of common occurrence^2^. While major depressive disorder (*i*.*e*. depression) is indeed unique to humans, many of its symptoms may be modelled in animals. In this context, research on depression-like states (DLS) in animal models has revealed that a range of neurobiological, physiological, and behavioural features related to stress exposure and/or neuroimmune interaction are common between humans and other vertebrates. In particular, a robust association between stress induced behavioural inhibition, brain serotonergic function and reduced neural plasticity and neurogenesis has been shown to be conserved throughout the vertebrate lineage^5–11^.

Taken together, the above observations suggest a biological background for depressed states and behaviour that is conserved by evolution. Empirical data directly quantifying potentially adaptive outcomes of behaviour associated with DLS are to our knowledge still lacking. Studies on social competence, which can be defined as an individual’s ability to use social information to optimise its social behaviour^12,13^, propose that subordinate animals have the capacity to adapt their behavioural output to novel social contests in order to reduce energetic expenditure and lower risk injuries, by displaying in general more submissive behaviours (such as behavioural inhibition, particularly under increased dominance threat^12^). In this context, several authors within evolutionary biology have proposed that DLS is an outcome of evolved mechanisms that contributed positively to reproductive success in ancestral environments^14–18^ and that a mismatch between the historic and current environment leads to normally adaptive responses overriding self-correcting tendencies of emotional mechanisms, and this leads to pathologies^15,16^. In agreement with this, we recently showed a potential evolutionary background for the DLS associated with chronic stress in Atlantic salmon (*Salmo salar* L.), in that serotonin-mediated behavioural inhibition may have evolved in vertebrates to minimize further stress in already exposed and vulnerable individuals^19^. In other words, DLS-associated behaviours may serve as a coping mechanism to threatening circumstances where flight is impossible^20,21^.

Here we address the possible benefits of behavioural-inhibition in a comparative model for social stress (commonly used to study DLS mechanisms) in the salmonid rainbow trout (*Oncorhynchus mykiss*). Previously it has been shown that subordinate fish under social stress show many neuroendocrine and behavioural alterations reminiscent of mammalian models of DLS, including behavioural inhibition^22^, a chronic  neuroendocrine stress response^23–25^, altered serotonergic function^26,27^, and reduced pallial neurogenesis^11^. In the present study, we show that behavioural inhibition in subordinate trout is reversed by the fast-acting antidepressant ketamine and discuss the implications of the resulting change in behaviour. Specifically, we found that a ketamine-induced increase in activity led to a higher amount of received aggression from dominant individuals. Given the multitude of negative outcomes from receiving aggression from dominant conspecifics, this observation implies that it is indeed beneficial to display behavioural inhibition in risky environments.

## Materials and Methods

### Ethics statement

This work was approved by the Norwegian Animal Research Authority (NARA), following the Norwegian laws and regulations controlling experiments and procedures on live animals in Norway (permit number 6156 granted Feb, 2014).

### Experimental animals and design overview

Rainbow trout from the commercial strain Aquagen AS were obtained from the facilities at The Norwegian University of Life Sciences (NMBU). Fish were reared in indoor tanks (Ø = 3 m, volume = 7 m^3^) on a 24 hour light regime at ambient temperatures (59.6663° N, 10.7679° E). After transfer to the aquaria facility at the department of Biosciences, University of Oslo, all fish were held in two indoor tanks (250 × 100 × 50 cm). They were acclimated to a 12:12 light/dark regime and monitored for signs of sickness and stress for a minimum of 4 weeks. All tanks were aerated and continuously supplied with dechlorinated Oslo tap water (pH 7.2–7.5) at ambient temperature (February-May 5–9.2 °C) throughout the experiment. For a detailed description of the water ion content, see Schjolden *et al*.^28^. Fish were fed once a day with 3 mm commercial food pellets (Skretting, Norway).

We first conducted a pilot study in order to find the lowest ketamine dose which resulted in increase activity. We then treated chronically stressed socially subordinate fish with ketamine before subjecting them to a novel dominance contest. For all experiments, fish were held in eight aquaria tanks (100 × 50 × 50 cm, volume = 250 L), which were divided into four equally sized compartments by opaque PVC walls and three sides of the aquaria were covered in black plastic. All tanks were continuously aerated and supplied with de-chlorinated tap water and kept at ambient temperature (March-May 5–9.2°C).

A waterproof closed-circuit camera system (Colour CCD cameras, IR- YC-25V, with a 3.6 mm lens) controlled by MSH video client software, was used to record behavioural interactions. Cameras were placed approximately 70 cm in front of each tank.

#### Experiment 1: Behavioural effects of ketamine

In order to characterise the response to ketamine in rainbow trout, a dose response experiment was conducted over two rounds. We diluted an initial concentration of 50 mg/mL ketamine (Ketalar, Pfizer, USA) with physiological saline into four different concentrations (Table 1) which were based upon reported ketamine effects in mammals and zebrafish studies^29–31^.

**Table 1:** Number of fish used for each Ketamine concentration in the dose-response experiment.

Dose	Number of fish (*n*)
O mg/kg (control)	16
5 mg/kg	20
10 mg/kg	12
15 mg/kg	8
25 mg/kg	8

The fish were anesthetized (to deep plane anaesthesia) in 50 mg/L tricaine methanesulfonate (MS-222, Sigma Aldrich, St. Louis, MO, USA), buffered to a pH of 7.2, weighed (Mean ± SD: 207.4 ± 55.8 g) and immediately injected intraperitoneally (*i*.*p*.) with a volume based on their total mass (*e*.*g*. a 100 g fish was injected with 100 μl) with either a saline solution containing one of the ketamine concentrations, or sham injected with physiological saline only and placed in individual compartments. Initially, ketamine doses of 5, 15 and 25 mg/kg, were tested on eight fish at a time for each concentration. Prolonged immobility and death were observed at the higher dose of 25 mg/kg (see results section). Therefore, during the second round, fish were tested with only the 5 or 10 mg/kg doses. Sham-injected control fish were included in both experimental rounds (see Table 1 for number of fish tested per dose). Fish were video recorded for 60 min (starting at 10:00 every day) for a period of 4 days, immediately after the ketamine/sham injection. Routine activity was analysed (*i*.*e*. time spent swimming over a minimum of one body length) for a total of 10 min, for a period of 4 days (injection day + the 3 subsequent following days).

#### Experiment 2: Social stress experiment

Fish were anesthetized (to deep plane anaesthesia) in a bath of 50 mg/L MS-222 (buffered to a pH of 7.2) before they were weighed and transferred to individual compartments. The experiment was carried out in two rounds due to the number of available aquaria at the research facilities. Two experimental conditions were established: i) isolated group consisting of small fish (*n* = 8; mean mass ± SD:158 ± 14 g, *i*.*e*. 4 fish per round) and ii) social stress group consisting of small (*n* = 24; mean mass ± SD:152 ± 16 g, *i*.*e*. 12 fish per round) and large (*n* = 24; mean mass ± SD: 266 ± 25 g, *i*.*e*. 12 fish per round) fish. During the 12-day acclimation period, fish were fed approximately 0.5% of their bodyweight in dry pellets once a day. The experiment started when all fish exhibited active feeding behaviour and consumed at least 50% of their food.

**Figure 1:**
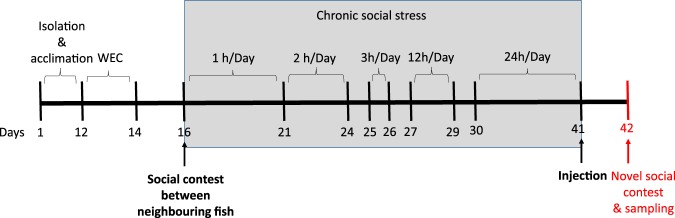
Schematic representation of the experimental timeline. After the acclimation period, from days 12 to 14 all experimental fish were subjected to a winner effect conditioning (WEC) paradigm in which all fish met a smaller intruder for 1 hour/day. At day 15 all fish were subjected to a dominance dyadic contest with their neighbouring fish (*i*.*e*. a small *vs*. a big fish). From day 16 up to day 41, the chronic social stress regime was conducted by exposing the subordinate small fish to their larger dominant conspecific neighbour in an increasing amount of time per day (hours per day given on figure). On day 41, the small subordinate fish were injected with ketamine or with saline water (sham) before exposing them to a larger novel conspecific for 1 hour before sampling.

Immediately after acclimatisation, all fish from the social stress group were subjected to a winner effect paradigm. This was done to ensure that all experimental fish had prior experience with winning in social contests. We introduced a smaller fish (approximately 33% smaller) to each fish for a period of 1 h during three consecutive days. The intruder fish were smaller in order to create an asymmetric dyadic contest in which bigger fish would win the dominance contest^32–34^, promoting a winner-effect (*i*.*e*. increased tendency for previously dominant fish to win subsequent dominance contests^32,33^). After three consecutive days of the winner effect conditioning, all experimental fish were paired for an asymmetric dyadic contest (one small *vs*. one big) with their neighbouring conspecific for 26 days. In order to avoid substantial injuries, the interaction time was gradually increased between neighbouring conspecifics throughout the experiment (for details see Fig. 1), until day 30, when both fish were left to interact together during the whole day for 10 consecutive days. Throughout this period, the aggressive interactions were video recorded and closely monitored. Social interactions were stopped if subordinate individuals showed signs of physical damage or extreme inhibition (*i*.*e*. remaining motionless on the bottom for more than 6 hours after social interactions). Notably, another reason why we conducted the winner effect conditioning is that it has been shown that when previous dominant animals become subordinates, they have a higher tendency to display a more pronounced behavioural inhibition^35^. In other words, by exposing small previously dominant fish to constant aggression by a larger dominant conspecific, we maximised the probability for subordinates to display DLS-type behaviour. On day 41, fish were isolated once more and the subordinates were injected with a single dose of 5 mg/kg ketamine (*n* = 12, *i*.*e*. 6 per round) or a sham (*n* = 12 *i*.*e*. 6 per round) solution (double-blinded) and left to recover overnight. At this stage, two ketamine injected fish had to be removed from further study due to aversive effects to the injection treatment. The fish in the isolated group were maintained in isolation throughout all this period and left undisturbed except for feeding and husbandry routines, until the injection day (*i*.*e*. day 41). The isolated fish were all sham injected on day 41 and left to recover until the next day (one fish had to be removed due to aversive effects to the injection treatment). On day 42 a one-hour social interaction (*i*.*e*. asymmetric dyadic contest) was conducted and video recorded. This was done by exposing the chronically socially subordinate treated fish (ketamine and sham injected) as well as the isolated sham injected fish to a larger novel conspecific (approximately 35% bigger to subordinates/isolated fish) for 20 min. After this interaction, the treated fish were immediately euthanized and sampled.

### Sampling

The fish were euthanized in a bath containing a lethal overdose of MS-222 (1 g/L, buffered to a pH of 7.2), which caused opercular movements to stop within 20 s. Fish were weighed, and a blood sample was taken from the caudal vessel with 23 G 1 ml syringes containing the anticoagulant ethylene diamine tetra acetic acid (EDTA). Following centrifugation for 5 min at 9.289 g and 4 °C, plasma samples were frozen and stored at −80 °C for later analysis.

### Quantification of behaviour: Video analysis

#### Experiment 1: Behavioural effects of ketamine

The amount of time spent moving (*i*.*e*. time spent swimming over a minimum of one body length) was used as a proxy for activity level and was calculated for all individuals for a period consisting of ten min per day for a total of four days.

#### Experiment 2: Social stress experiment

During the chronic social stress period (*i*.*e*. during 25 days, see Fig. 1 for further details), aggressive interactions between neighbouring fish were filmed, and observed although not quantified. All small fish became subordinate and exhibited the stereotypical subordinate profile, *i*.*e*. suppressed aggressive behaviour, fleeing from the dominant and receiving unidirectional aggressive acts from the larger dominant fish^36^. The final social interaction between the intruder subordinate fish (ketamine treated and sham control) and the novel larger conspecific was video recorded for a total of 60 min (out of which 20 min were analyzed) with a Sony HDR (CX320E) camera, which was placed on a tripod approximately 70 cm in front of the aquaria. In the final dyadic contest, fight duration (*i*.*e*. amount of time fish spend fighting before dominance is established) was quantified. Notably, a majority of the small chronically subordinate fish did not challenge the bigger fish for dominance and therefore the fight duration was set to 1 s. That is, once the bigger fish became aware of the smaller intruder it attacked straight away and was not challenged by the smaller fish. In 7 cases (4 control, 1 ketamine and 2 isolated) the small fish remained completely immobile at the tank bottom and the fight duration was set to 0 s. In addition, the number of received attacks (*i*.*e*. charging, biting or nipping and chasing) after contest resolution were quantified and pooled together in order to calculate the total number of aggressive acts received by each subordinate fish. Furthermore, activity levels were quantified during this period as explained above.

### Blood plasma analysis

Blood plasma samples were analysed for ketamine content to corroborate that treated fish had ketamine in their system. Plasma samples from each group (*i*.*e*. ketamine or sham injected) were pooled together (*i*.*e*. 5 individual samples were pooled together, making a total of 2 samples per group) for methodological and technical reasons. The ketamine analysis was performed at the Department of Clinical Pharmacology, St. Olav University Hospital, Trondheim, Norway, with a liquid chromatography mass spectrometry (LC-MS) method developed at the laboratory. In brief, ketamine was extracted from 1.0 mL plasma by a liquid-liquid method adding griseofulvin as an internal standard and separated on a Zobrax SB-C18 (150 × 4.6 mm) column using an LC-MSD 1100-system (Agilent, Palo Alto, California, USA). The LC-MS was operated in the positive ionization mode, using a mass transition of m/z 238.1 > 125.1 for ketamine and 352.7 for griseofulvin. The limit of quantification was 10 nM, and the method was linear at least up to 4000 nM.

### Statistical analysis

A repeated-measures analysis of variance (ANOVA) was used to compare the amount of time spent moving (*i*.*e*. activity level) with treatment (all ketamine doses and control) and time (days 0, 1, 2 and 3) as independent variables. Planned contrast effects tests were conducted between each dose and control. In addition, a significant effect between treatment and control (*i*.*e*. the 5 mg/kg dose only) was followed by planned contrast effect tests between treatment and control within each day (*i*.*e*. at day 0, 1, 2 and 3), but not between days. Received aggression and activity levels after the final dyadic contest were analysed by means of a two-way ANOVA with treatment and weight as factors. This was followed by a Tuckey post-hoc test in order to identify significant differences between the 3 groups. The models were assessed by their capacity to explain the variability. Before final acceptance of the model, diagnostic residual plots were examined to ensure that no systematic patterns occurred in the errors (*e*.*g*. fitted values *vs*. observed values and q-q plots). Furthermore, Spearman’s correlation analysis was conducted in order to study the relationship between received aggression and activity level in all groups.

## Results

### Experiment 1: Behavioural effects of ketamine

**Figure 2:**
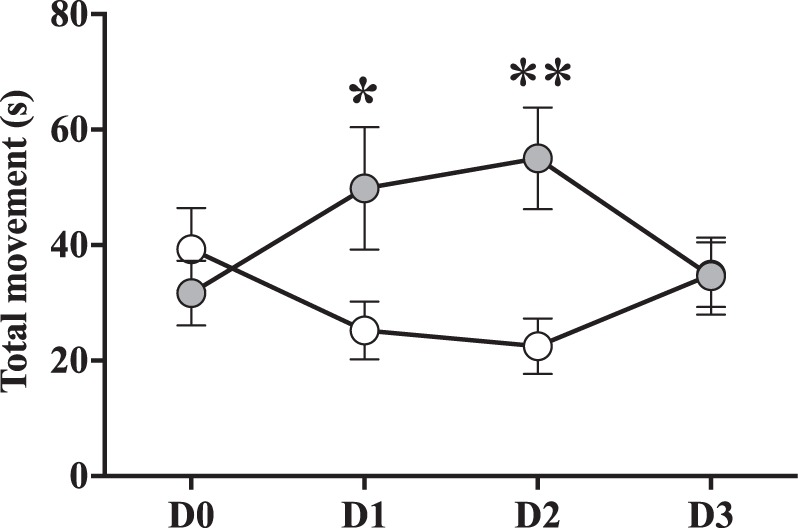
Ketamine dose experiment. Mean (±SEM) activity levels (measured as total time spent moving over one body length during 10 min) immediately after treatment with a 5 mg/kg Ketamine dose *vs*. a sham saline water (control) at day 0 (day of the injection) and the 3 following days. Repeated ANOVA statistics: Treatment *F*_(19,215)_ = 3.75, *p* = **0**.**006**, Time *F*_(19,215)_ = 1.5, *p* = 0.21, with * indicating significant post-hoc test differences between groups per day.

We found a dose-dependent effect of ketamine on the general activity levels of rainbow trout. There was a significant effect of treatment (*F*_*(19*,*215)*_ = 3.75, ***p*** = **0**.**006**) but not time (*F*_*(19*,*215)*_ = 1.5, *p* = 0.21) on fish activity levels, with the 5 mg/kg dose showing an increase (***p*** = **0**.**02**) in activity compared to control fish. In addition, there was a tendency (*p* = 0.07) for the 25 mg/kg to decrease activity levels, compared to saline-treated controls. Post-hoc analysis between the 5 mg/kg dose and control groups at all time points showed that ketamine fish had a significant increase in activity at days 1 (***p*** = **0**.**02**) and 2 (***p*** = **0**.**002**), compared to controls (Fig. 2). Notably, we found that the injection procedure resulted in an immediate (*i*.*e*. Day 0), moderate, non-significant reduction in movement on all ketamine-treated fish. Furthermore, the higher ketamine dose (*i*.*e*. 25 mg/kg) had an undesired anaesthetic effect, which resulted in the death of 4 out of 8 fish in this group.

### Experiment 2: Social stress experiment

In general, during the final dyadic contest there was no challenge from the small chronically subordinate individuals towards the bigger fish. That is, after being introduced into the bigger conspecific’s tank, the small fish displayed no aggressive behaviour and only received aggression from the larger conspecific. The average (±SEM) fight duration was 1 ± 0.4 s, 18.8 ± 18 s and 35.9 ± 17.7 s for control, ketamine and isolated fish, respectively. Notably, while only one subordinate fish from the control and one from the ketamine groups challenged the bigger novel conspecific, five out of seven fish from the isolated group challenged the bigger fish for dominance.

**Figure 3:**
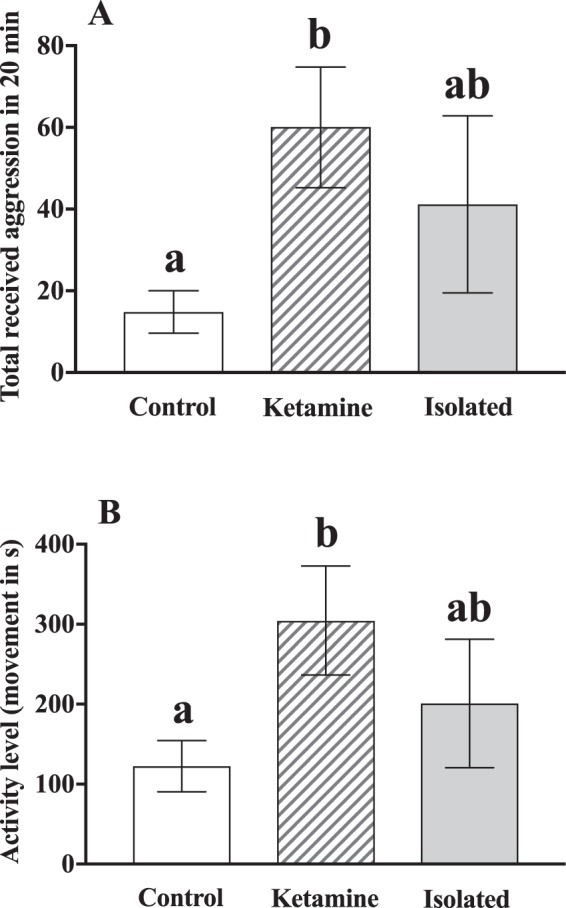
Asymmetric dominance social contest between chronic socially stressed subordinate and a novel larger conspecific. (**A**) Total amount of received aggression accrued during 20 min by subordinate ketamine-treated, control and isolated fish. ANOVA statistics: Treatment *F*_(3,25)_ = 3.85, *p* = **0**.**03**, Weight *F*_(3,25)_ = 1.14, *p* = 0.3 (**B**) Total amount of activity level (measured as total time spent moving over one body length) over 20 min by subordinate ketamine-treated, control and isolated fish. ANOVA statistics: Treatment *F*_(3,25)_ = 3.74, *p* = **0**.**04**, Weight *F*_(3,25)_ = 2.8, *p* = 0.1. Small letters indicating significant post-hoc test differences between groups.

**Figure 4:**
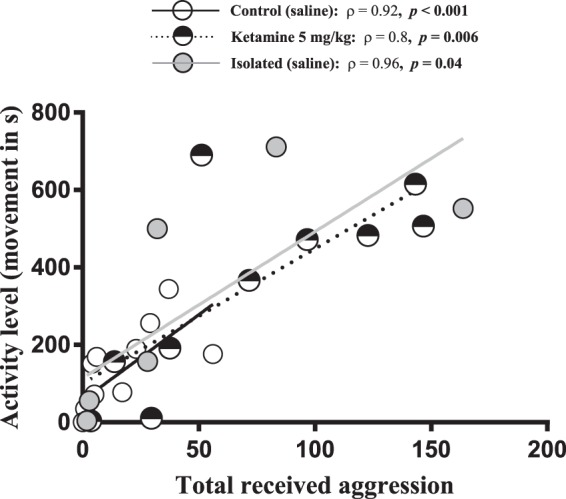
Correlation analysis showing the relationship between activity level (measured as total time spent moving over one body length during 20 min) and amount of received aggression during 20 min for subordinate ketamine-treated, control and isolated fish during an asymmetric dominance dyadic contest with a novel larger conspecific. Spearman’s correlation analysis statistics are given in the panel.

We found a significant effect of treatment on aggression (*F*_*(3*,*25)*_ = 3.85, ***p*** = **0**.**03**) and activity levels (*F*_*(3*,*25)*_ = 3.74, ***p*** = **0**.**04**) but not weight (aggression *F*_*(3*,*25)*_ = 1.14, *p* = 0.3 and activity *F*_*(3*,*25)*_ = 2.8, *p* = 0.1) for Ketamine, control and isolated fish. Specifically, subordinate ketamine-treated fish moved more (***p*** = **0**.**03**) and received more aggression (***p*** = **0**.**03**) than saline injected controls when exposed to a novel larger conspecific (Fig. 3). However, no significant differences were found between isolated and control (*p*_*activity*_ = 0.4 and *p*_*aggression*_ = 0.3) or between isolated and ketamine fish (*p*_*activity*_ = 0.5 and *p*_*aggression*_ = 0.6). There was a highly significant correlation between received aggression and activity levels for control (ρ = 0.92, *p* < 0.001), ketamine (ρ = 0.8, *p* = 0.006) and isolated (ρ = 0.96, *p* < 0.001) groups (Fig. 4).

The ketamine treated fish (5 mg/kg) had between 13.4–36.8 nM ketamine in their blood plasma, while no ketamine was found in control or isolated fish.

## Discussion

We here present empirical evidence suggesting that a depression-like behavioural output (*i*.*e*. behavioural inhibition) has a potential beneficial effect in a risky social environment (*i*.*e*. under threat from a previously unknown dominant individual). This is in agreement with the social rank hypothesis of depression, which proposes that DLS may help individuals cope with threatening circumstances where flight/escape is impossible, such as for subordinate individuals in social hierarchies^20^. Notably, it is important to clarify that while behavioural inhibition may be an adaptive response under these conditions, a depression-like state is not necessarily a response chosen by an individual, but instead a condition that results from a normally adaptive response overriding self-correcting tendencies of emotional mechanisms, which in turn leads to pathology^15,16^.

Agonistic interactions during a dyadic social contest typically leads to the establishment of a dominant-subordinate relationship/hierarchy in several animal species, in which subordinates suppress their aggression and retreat from dominant individuals, *i*.*e*. they show a general inhibition of normal behaviour^22,24^. Behavioural inhibition in subordinate animals can be viewed as a passive coping response serving to avoid costly interactions with aggressive dominant individuals^37^, as constant high levels of received aggression can be detrimental. In this context, social stress in fish has been proposed to be a good model for the study of DLS^19,38–40^. It has been hypothesised that brief periods of behavioural inhibition may limit predation, injuries and exhaustion during stressful situations in fish^15,19,41^. To our knowledge, the current study constitutes the first empirical support for such an evolutionary hypothesis on the biological background for a DLS-associated behaviour.

Ketamine has classically been used as an anaesthetic, but recent studies have determined that sub-anaesthetic doses of ketamine increase activity and that it may be used as a fast-acting antidepressant drug^29,30,42^. In agreement with our results, ketamine treatment at sub-anaesthetic doses has been found to increase activity levels in several animal species. For example, it has been reported that sub-anaesthetic doses of ketamine increase movement in isolated zebrafish^30^. Furthermore, ketamine-induced increase in activity has been well documented in mammals *e*.*g*.^29,43,44^. Our data show that in rainbow trout a sub-anaesthetic single dose of ketamine was enough to increase activity levels in trout already one day after injection. During encounters with a large, aggressive conspecific ketamine-treated subordinate fish also showed increased activity levels, and there was a significant correlation between activity and received aggression.

Salmonids establish dominance hierarchies by engaging in social contests. These contests are characterized by an initial phase in which the fish display with their fins raised and circle each other. Following this, the contest becomes more aggressive as both fish resort to nips, bites, charges and chases in order to win the dominance contest. However, once they achieve contest resolution, there is only unilateral displays of aggression from the dominant towards the subordinate. At this point, the subordinate may limit the amount of received aggression by either fleeing or remaining motionless at the bottom, often displaying a darker coloration^36,45^. Interestingly, size discrepancies affect the outcome of fish social contests and individuals having a 20% or more size advantage on their opponents often gain dominance over their smaller conspecifics^34,46,47^. In our experiment, we found that when small chronically subordinate fish encountered a bigger novel conspecific (approximately 35% bigger), there was mainly unidirectional aggression from the bigger fish towards the smaller one. That is, the smaller fish did not challenge the bigger fish for dominance and instead tried to either flee or remain motionless at the bottom. However, ketamine fish were unable to remain completely motionless and therefore incurred higher levels of aggression. Interestingly, 5 out of 7 isolated fish challenged the bigger conspecific, which lead to a longer fight duration and a higher variation in amount of received aggression after contest resolution. In addition, two fish remained immobile throughout the whole experimental period and therefore did not incur any aggression from the bigger fish. Thus, it would appear that previous social encounters lead to a more uniform behaviour during contest. We maintain that also this aspect of the DLS-associated behaviour may incur a fitness advantage, particularly when the size difference is so large that a small individual does not have a chance of winning a social contest. The behavioural inhibition conceivably allows these individuals to conserve energy (by reducing activity levels and avoiding the fight for dominance) and avoid aggression. Ketamine injection would seem to reverse this response to social subordination and increase activity levels, but without increasing variability to the level seen in individuals without prior social experience. This is in agreement with the literature on social competence, which proposes that animals within a social context will optimise their behaviour based on the available information and previous experiences^12,13^. Even though it is highly adaptive for individuals to know when to show submissive behaviour, being subordinate is costly for the individual, as it is associated with reduced reproduction, low activity levels, reduced growth and suppressed aggression and feeding^10,37,48,49^. In this context, it is important to consider that even though behavioural inhibition may have negative consequences for the fitness of subordinate individuals in the long-term, this behavioural response (shown by all subordinates to a certain degree) has not been removed by selection, particularly since it appears to be an important component of social group living^12,13,50^. Subordinate individuals in groups with “despotic” (in which dominance is attained by aggression and intimidation) stable hierarchies, show the greatest physiological indices of stress^51^ and it is therefore necessary for these individuals to adjust their behaviour, such as adopting submissive behaviour, in order to reduce their stress load. Importantly, we previously found that DLS fish in aquaculture farms were characterized by behavioural inhibition and elevated basal serotonergic and cortisol levels and that these individuals were unable to respond further with a serotonergic response to acute stress, although a cortisol response was maintained^19^. This increase in cortisol but not serotonin leaves individuals vulnerable to the long-terms effects of cortisol in the brain, and thus gives a physiological explanation to the behavioural profile associated with DLS fish. Presently, we show that a DLS behavioural response (behavioural inhibition) is beneficial in reducing aggression levels from dominant conspecifics, compared to antidepressant-treated individuals. Further research in animal models of DLS are needed to shed light on the possible adaptive value of behavioural inhibition, especially with regards to whether the behavioural profile is reversible should circumstances change.

To our knowledge, our results constitute the first empirical evidence that the stress induced behavioural inhibition characterizing DLS may confer an adaptive advantage under threat from a dominant conspecific. We believe that these findings should encourage further exploration of the evolutionary background for stress-induced behavioural inhibition. Such knowledge may be pivotal in advancing the development of new approaches to stress-induced neurobiological pathologies and may ultimately facilitate a shift in focus to the medical ideal of prevention rather than cure.

## Supplementary information

Data set 1

## Data Availability

All relevant data are within the paper and its supplementary material.
